# Bromodomain and Extraterminal Proteins as Novel Epigenetic Targets for Renal Diseases

**DOI:** 10.3389/fphar.2019.01315

**Published:** 2019-11-08

**Authors:** Jose Luis Morgado-Pascual, Sandra Rayego-Mateos, Lucia Tejedor, Beatriz Suarez-Alvarez, Marta Ruiz-Ortega

**Affiliations:** ^1^Cellular Biology in Renal Diseases Laboratory, IIS-Fundación Jiménez Díaz, Universidad Autónoma Madrid, Madrid, Spain; ^2^Red de Investigación Renal (REDinREN), Madrid, Spain; ^3^Vascular and Renal Translational Research Group, Institut de Recerca Biomèdica de Lleida (IRBLleida), Lleida, Spain; ^4^Translational Immunology Laboratory, Health Research Institute of the Principality of Asturias (ISPA), Hospital Universitario Central de Asturias, Oviedo, Spain

**Keywords:** BET proteins, renal injury, inflammation, fibrosis, chronic kidney disease, epigenetic modifications

## Abstract

Epigenetic mechanisms, especially DNA methylation and histone modifications, are dynamic processes that regulate the gene expression transcriptional program in normal and diseased states. The bromodomain and extraterminal (BET) protein family (BRD2, BRD3, BRD4, and BRDT) are epigenetic readers that, *via* bromodomains, regulate gene transcription by binding to acetylated lysine residues on histones and master transcriptional factors. Experimental data have demonstrated the involvement of some BET proteins in many pathological conditions, including tumor development, infections, autoimmunity, and inflammation. Selective bromodomain inhibitors are epigenetic drugs that block the interaction between BET proteins and acetylated proteins, thus exerting beneficial effects. Recent data have described the beneficial effect of BET inhibition on experimental renal diseases. Emerging evidence underscores the importance of environmental modifications in the origin of pathological features in chronic kidney diseases (CKD). Several cellular processes such as oxidation, metabolic disorders, cytokines, inflammation, or accumulated uremic toxins may induce epigenetic modifications that regulate key processes involved in renal damage and in other pathological conditions observed in CKD patients. Here, we review how targeting bromodomains in BET proteins may regulate essential processes involved in renal diseases and in associated complications found in CKD patients, such as cardiovascular damage, highlighting the potential of epigenetic therapeutic strategies against BET proteins for CKD treatment and associated risks.

## Introduction

Chronic kidney disease (CKD) is characterized by a loss of the nephron functionality. This pathological situation is triggered by an inflammatory response that damages the resident cells of the kidney, thus activating several intracellular mechanisms, which amplifies the renal damage and finally leads to fibrosis. Nowadays, the elevated presence of risk factors such as obesity, hypertension, diabetes, and aging increases the prevalence of CKD; currently, CKD is estimated to affect approximately 10% of the EU population. When CKD progresses towards the end stage (ESRD), patients require a replacement therapy. Patients who do not receive a kidney transplant require dialysis treatment (64% of patients), resulting in an annual cost of €15 billion to EU health systems. Furthermore, CKD has been established as public-health priority due the high risk of cardiovascular complications associated with the disease. Nowadays, therapeutic options against CKD are limited, hence the value of novel therapeutic options that can prevent the initial development of the pathology or its progression. Currently, there is several evidence of how the environmental modifications or altered pathological factors that appear during CKD (oxidative stress and inflammation, among others) could be induced by epigenetic modifications or changes. In renal pathology, the most widely studied epigenetic alterations are DNA methylation, histone modifications (acetylation, phosphorylation, methylation, etc.), and changes in miRNAs levels (Morgado-Pascual et al., 2018). These epigenetic changes regulate the transcription of many genes involved in the inflammatory and immune response, and other relevant process involved in renal pathology and in cardiovascular-related complications (Keating and El-Osta, 2013; Morgado-Pascual et al., 2018).

The relevance of these investigations has led to the growing development of small molecules targeting enzymes responsible for “writing,” “reading,” or “erasing” of the epigenetic modifications. Among them, the bromo- and extraterminal (BET) proteins are epigenetic “readers,” which recognize and bind to the acetylated lysine in histones and other proteins. In this review, we will discuss the role of BET proteins in renal pathology and associated diseases such as cardiovascular damage, highlighting its potential role as a therapeutic target for CKD treatment.

### BET Proteins

**Figure 1:**
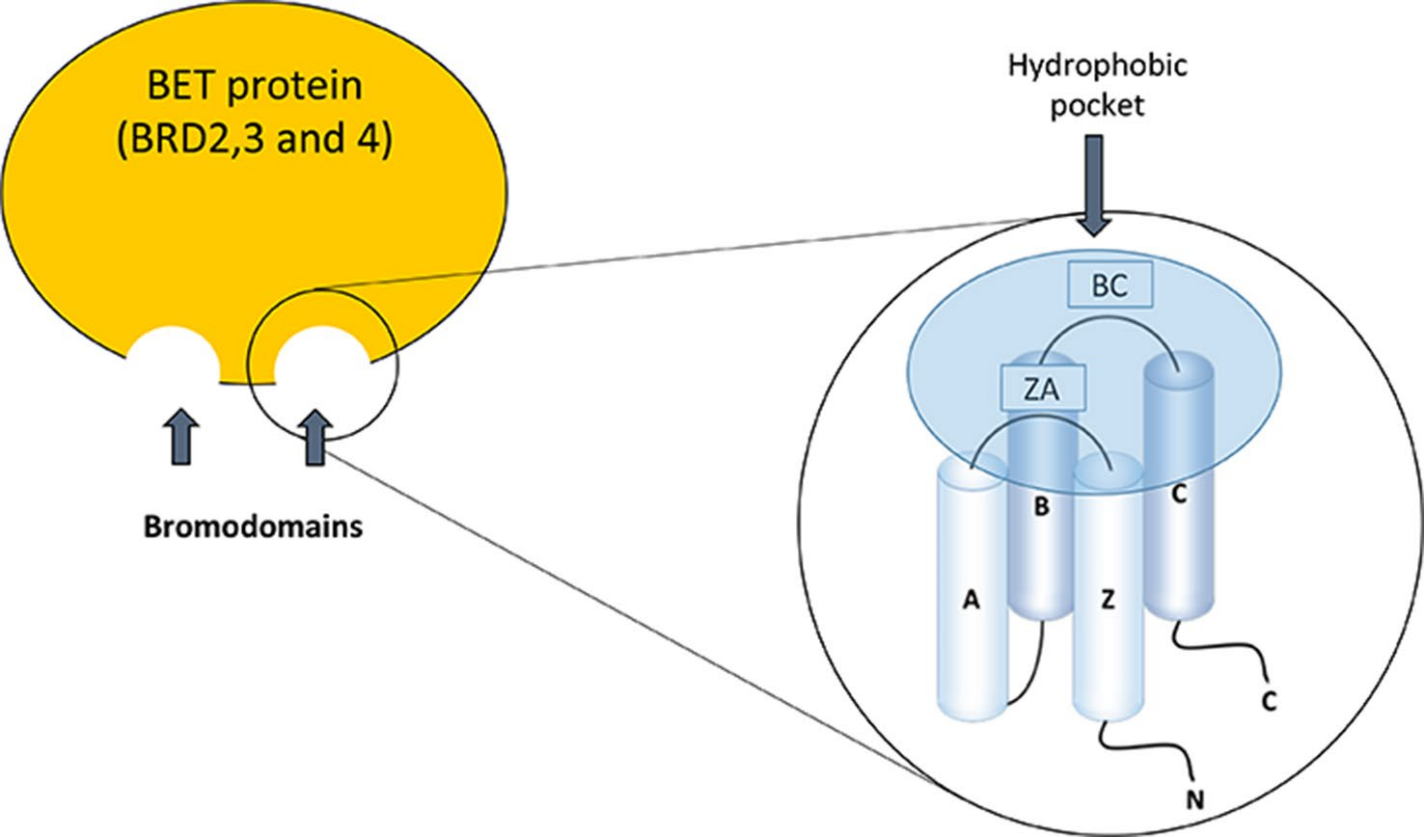
Bromodomain structure in the bromodomain and extraterminal (BET) family proteins.

The BET family consists of four members; BRD2, BRD3, and BRD4, which are ubiquitously expressed, and BRDT, whose expression is limited to the male germ cell. These proteins are located in the nucleus of the cell, but the functional differences between them are not well defined (Markowski et al., 2017; Radwan et al., 2017). Bromodomains (BD) were discovered for the first time in studies on the gene Brama in *Drosophila* (Haynes et al., 1992) and there are now described at least 61 types of bromodomains encoded in the human genome. The structure of the bromodomains is formed by four helix packed with two loops of different length between their helices, BC and ZA (**Figure 1**). The hydrophobic pocket, formed by the BC and ZA loops, is coated with residues of Tyr97, Asp140, Val752, Ala757, Tyr760, Val763, Tyr802, and Tyr 809. The hydrophobic pocket is an ideal site for protein–protein interactions. It is located at one end of the packet of four helices, opposite the amino and carboxyl terminal zone of the protein (Jeanmougin et al., 1997). Bromodomains serve as regulators of the protein–protein interaction in different cellular processes such as transcription and chromatin remodeling (Sanchez et al., 2014). Furthermore, they are not only present in “reading” proteins, which are responsible for recognizing different epigenetic modifications (de la Cruz et al., 2005), but are also present in 46 different types of proteins such as acetyltransferases, chromatin-associated proteins, transcriptional coactivators, and methyltransferases (Dhalluin et al., 1999).

**Figure 2:**
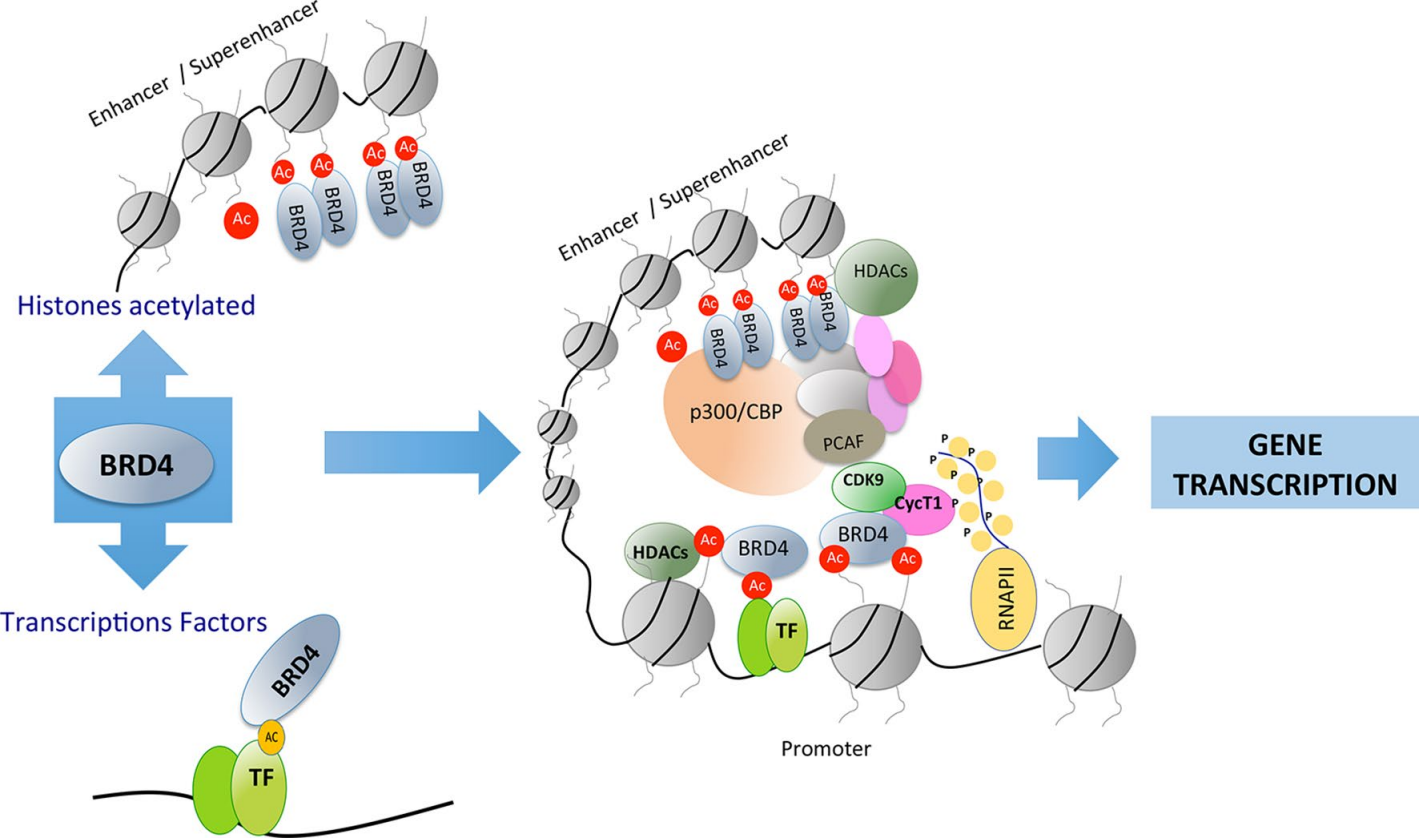
Bromodomain and extraterminal (BET) proteins recognize acetylated residues in histones or in other proteins such as transcription factors. BET proteins can recruit transcription factors in distant areas from the promoter of genes involved in different cellular processes.

The structure of BET proteins includes a tandem of two conserved N-terminal bromodomains (BD1 and BD2), an extraterminal domain (ET), and a c-terminal domain (CTD) (**Figure 1**). The BDs are amino acidic hydrophobic regions where protein–protein interaction occurs, and by this region BD interact as an epigenetic “reader” (Wu and Chiang, 2007; Wang et al., 2017). The ET domain is responsible of the recruitment of different proteins that are components of the transcriptional complex. The CTD is only possessed by two of the family members of BET proteins (BRD4 and BRDT), and it is necessary for the recruitment of positive elongation factor (P-TEFb). BET proteins can bind to acetylated lysines of the histones located in the “super-enhancers” (DNA regions enriched with repressive acetylated H3K27 marks and RNA polymerase II) or promoter regions of the genes. After this binding, BET proteins, *via* CTD, participate in the recruitment of P-TEFb to the transcriptional complex (forming a heterodimer of CDK9 and cyclin T1 or T2). This union determines the degree of chromatin compaction, acting as a regulator of gene expression (Jang et al., 2005; Devaiah et al., 2012). In addition, BET proteins *via* bromodomain can also interact with acetylated lysine residues in other proteins such as transcription factors, regulating their function (Wang et al., 2018) (**Figure 2**).

**Figure 3:**
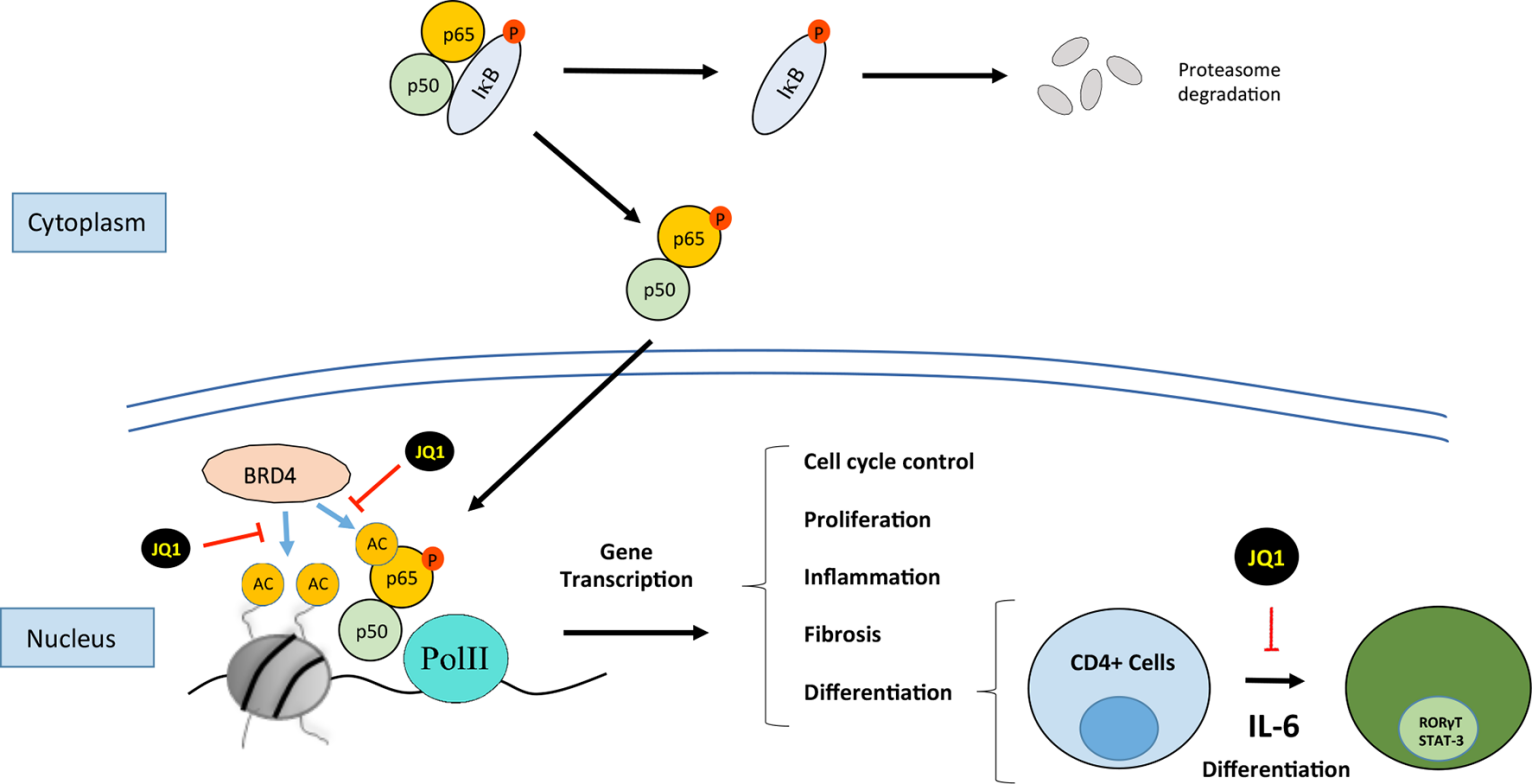
Bromodomain and extraterminal (BET) proteins regulate cellular processes such as cell cycle, proliferation, differentiation, inflammation, and fibrosis. JQ1 binds to BRD4, this situation not allows the recruitment of p65 and finally it is ubiquitinilated and degraded by the proteasome. BRD4 interacts with NF-kB to induce the expression of genes associated with processes such as inflammation or TH17 immune response.

BET proteins, which are epigenetic regulators of gene transcription, are strongly implicated in the regulation of cell growth, differentiation, and inflammation (**Figure 3**). These proteins are located in the nucleus and regulate many cellular activities including gene transcription, DNA replication, cell-cycle progression, and, therefore, participate in tumor development, infections, autoimmunity, and inflammation (Boehm et al., 2013; Xu & Vakoc, 2014). Although some research has been carried out the functional differences between the distinct BET proteins are not well defined yet.

#### BRD4 the Most Studied BET Protein

The most comprehensively characterized BET member is BRD4 (originally named mitotic protein associated with chromatin or MCAP). BRD4 was identified in 1988 in studies on mammals as a coactivator protein involved in gene transcription (Huang et al., 2009). Since then, several studies have demonstrated the role of BRD4 in several steps of the transcription hierarchy. BRD4 was firstly described as a proliferative target in a cancer study of nuclear testis protein (NUT) midline carcinoma caused by the nuclear translocation of the BRD4-NUT oncogene product (Wang and You, 2015). The product of this gene acts by driving the cell in a permanent proliferative phase (French et al., 2008; Filippakopoulos et al., 2010). Initial studies described that BRD4 recruits the p-TEFb complex to the promoter region of genes to activate transcription (Barboric et al., 2001; Jang et al., 2005; Devaiah et al., 2012). The ET domain of BRD4 independently recruits transcriptional activators such as NSD3 (a histone methyl transferase), JMJD6 (a histone arginine demethylase), and CHD4 (the catalytic subunit of the NuRD chromatin remodeler) or P300 (acetyl transferase) (Taniguchi, 2016). Subsequent studies reported that BRD4 binds to acetylated histones in the enhancers or promoter regions of inflammatory genes (Lovén et al., 2013). In addition, BRD4 has intrinsic kinase activity and phosphorylates the Pol II CTD at the Ser2 position; these results indicate a clear role for BRD4 in the regulation of transcription (Devaiah et al., 2012). BRD4 can also bind to acetylated residues in other proteins besides histones. Among these proteins, transcription factors have special relevance, as they are an additional mechanism by which BET can regulate gene transcription (Wu and Chiang, 2007; Alpatov et al., 2014). The most widely investigated is the transcription factor nuclear factor-κB (NF-κB). Studies in cancer have found that NF-κB is regulated by BRD4, acting as a coactivator, by binding to acetylated lysine-310 residue of the RelA NF-κB subunit (Huang et al., 2009). In cancer cells, the binding of BDR4 to RelA/NF-κB subunit blocks RelA ubiquitinilation and its subsequent proteasome-mediated degradation, leading to sustained nuclear NF-κB activation and, therefore, showing a mechanisms involved in aberrant cell proliferation (Wu et al., 2013; Zou et al., 2014).

### Targeting BET Proteins as a Potential Source of Novel Therapeutic Options.

The development of drugs targeting BET proteins has attracted special interest. The BET protein inhibitors (iBETs) compete for the bromodomain binding pocket and displace BET proteins from the binding to acetylated lysine residues located in histones. This interrupts the remodeling of chromatin and prevents the expression of certain genes, therefore regulating cellular responses (**Figure 3**).

#### BET Proteins in the Regulation of Cell Proliferation: Potential Therapeutic Role in Cancer and Proliferative Disorders

One of the earliest iBETs described was JQ1. This compound acts as pan-specific BRD4 inhibitor that binds to both BD1 and BD2 bromodomains in BRD4, therefore blocking its binding to acetylated lysine residues located in histones and other proteins (Maxmen et al., 2012). The first studies were done in experimental proliferative pathologies, including midline carcinoma and hematological malignances (Alqahtani et al., 2019). Initial studies showed that JQ1 exerted high antiproliferative responses associated with cell-growth arrest and senescence in experimental models of multiple myeloma (Delmore et al., 2011), and described that JQ1 mainly target c-Myc expression (Bandukwala et al., 2012). Since then, many preclinical studies have shown that JQ1 exerts beneficial effects in different proliferative disorders (Bandukwala et al., 2012; White et al., 2019), and in other pathologies, as it will be discussed later. Interestingly, in a murine model of polycystic kidney disease, JQ1 delayed cyst growth, by inhibiting c-Myc gene expression and cystic epithelial cell proliferation (Zhou et al., 2015). However, this iBET cannot be used in humans due to its pharmacological properties. Nowadays, there is a large battery of iBETs developed by diverse pharmaceutical companies. Most of them have been tested in different experimental malignances, and some of them currently tested on clinical trials (as it will be discussed latter).

### BET Proteins in Inflammation and Immune Responses in Renal Diseases

#### Potential Role of iBETS as Anti-Inflammatory Drugs *via* Inhibition of Proinflammatory Gene Expression

Many *in vitro* studies have found that BET proteins regulate the transcription of proinflammatory genes. BRD4 binds to acetylated histones in the enhancers or promoter regions of inflammatory genes (Lovén et al., 2013), as demonstrated for *IL-6, CCL-2*, and *CCL-5* in renal cells (Suarez-Alvarez et al., 2017). In activated macrophages, JQ1 and I-BET reduced LPS-mediated gene upregulation of cytokines (*TNF-α* and *IL-6*) and chemokines (*CCL-2*) (Nicodeme et al., 2010; Belkina et al., 2013), as observed in Raw 267.4 cells treated with JQ1 (Meng et al., 2014). In synovial fibroblasts obtained from rheumatoid arthritis patients, iBET-151 diminished their capacity to recruit immune cells by the inhibition of CXCL10 and CXCL11 (Klein et al., 2016). In human endothelial cells a novel iBET specific for BD2, apabetalone (initially named RVX-208), also reduced the gene expression of VCAM-1 and CCL2 induced by TNF-α or LPS (Jahagirdar et al., 2014). Using a whole-genome gene expression array, our group reported that in activated renal cells, JQ1 decreased TNF-α-mediated overexpression of several genes involved in the regulation of the inflammatory process and the immune response. These *in vitro* data suggested that iBETs could be used as anti-inflammatory drugs.

Anti-inflammatory properties of iBETs have been observed in different preclinical studies. Most of the studies have been done using the pan-specific iBET JQ1 in preventive therapeutic treatment (Liu et al., 2014; Zhang et al., 2015). In a collagen-induced experimental arthritis model, JQ1 induced attenuation in the arthritis severity score associated to lower serum levels of proinflammatory cytokines, including IL-1β, IL-6, IL-17, and IL-18 (Zhang et al., 2015). Another study in experimental periodontitis showed that JQ1 reduced bone destruction and decreased proinflammatory cytokine expression (IL-1β, IL-6, TNF-α, and IL-17) (Meng et al., 2014). In a model of experimental atherosclerosis in hyperlipidemic ApoE-deficient mice treatment with the BD2 inhibitor apabetalone reduced the aortic lesion formation, decreased circulating adhesion molecules (haptoglobin, VCAM-1, IL-18, SAP, and M1P1a) and reduced mRNA levels of proinflammatory cytokines (IL-6 and VCAM1) in carotid tissue (Jahagirdar et al., 2014). In different experimental models of renal damage, including unilateral uretheral obstruction (UUO), infusion of Angiotensin II (AngII) and immune-mediated glomerulonephritis, JQ1 inhibited proinflammatory gene expression associated to the diminution of the presence of inflammatory cells in the damaged kidneys. Moreover, by chromatin-immunoprecipitation experiments, we demonstrated that JQ1 displaces BRD4 binding to the acetylated-histone H3 in the promoter region of the proinflammatory genes IL-6, CCL-2, and CCL-5 (Suarez-Alvarez et al., 2017), showing a mechanism involved in these anti-inflammatory properties of iBETs in experimental renal diseases (**Figure 3**).

#### Potential Role of IBETS as Anti-Inflammatory Drugs in Renal Diseases *via* Inhibition of the Transcription Factor NF-κB

BET proteins can also bind to acetylated residues in transcription factors. The NF-κB pathway is a key transcription factor involved in the regulation of renal inflammation. In experimental and human renal diseases, the renal activation of the NF-κB pathway is associated with the overexpression of proinflammatory mediators, as initially described in human biopsies of diabetic nephropathy patients, showing colocalization of activated NF-κB and upregulation of *CCL2* mRNA expression levels (Mezzano et al., 2004). Many experimental investigations have described that NF-κB inhibition, by different strategies, including blocking specific components of this pathway, or indirectly by drugs used in the clinic to treat CKD patients, such as blockers of the renin angiotensin system, diminishes renal inflammation, and ameliorates disease progression (Sanz et al., 2010; Chung and Lan, 2011). The RelA NF-κB subunit can be acetylated in lysine 310 leading to its activation. As commented before, studies done in proliferative disorders have described that BRD4 binding to acetylated lysine-310 of RelA is essential to activate specific NF-κB target genes (Amir-Zilberstein et al., 2007; Huang et al., 2009; Zou et al., 2014). In an experimental diabetic model of renal damage in db/db mice, increased NF-κB was described and in podocytes stimulated with AGE, the treatment with the BET inhibitor MS417 suppressed acetylation of Stat3 and NF-κB. (Liu et al., 2014). Interestingly, we have observed that BET inhibition with JQ1 reduced RelA nuclear levels in several experimental models of renal damage and in TNF-α-treated renal cells, and thereby blocked NF-κB transcriptional activation and downregulated several NF-κB-controlled genes, including CCL-2 and IL-17A (Suarez-Alvarez et al., 2017), suggesting another mechanism contributing to the anti-inflammatory effects of iBETs in renal damage (**Figure 3**). Other iBETs also blocked NF-κB pathway and diminished renal inflammation. In the UUO model, treatment with I-BET151 (specific inhibitor against BRD2, BRD3, and BRD4) suppresses the phosphorylation and acetylation of NF-κB associated with a decrease in the number of CD68^+^ cells (macrophages) located in tubulointerstitium (Xiong et al., 2016). Moreover, I-BET151 also reduced the phosphorylation of STAT3 and the MAP kinases Erk1/Erk2 after UUO, but the direct effect of iBET in these pathways has not been demonstrated (Xiong et al., 2016). Interestingly, *in vitro* data showed that JQ1 specifically block active NF-κB, as shown by the degradation of RelA nuclear levels, without changing phosphorylated RelA levels in the cytosol (**Figure 3**). Moreover, the results of the DNA-array analysis showed that BET inhibition mainly downregulates proinflammatory genes, while genes controlling several NF-κB pathway components, such as the NF-kB subunits p50, RelA, RelB, IKB, and A20/TNFAIP3, were not modify (Suarez-Alvarez et al., 2017). Similar findings were described in the regulation of A20/TNFAIP3 (Huang et al., 2009). These results suggest that iBETs could be a unique pharmacological strategy to block the active NF-κB *via* the blockade of the interaction of BRD4 with nuclear acetylated-RelA. This strategy could be a more selective anti-inflammatory option, that other NF-κB targeting-drugs, such as kinase inhibitors or proteasome-targeted drugs.

#### Potential Role of iBETS as Anti-Inflammatory Drugs in Renal Diseases *via* TH17 Immune Response Modulation

Epigenetic regulation of cytokines and transcription factors has been shown to be important in directing lineage differentiation of CD4 T lymphocytes to Th1, Th2, Th17, or regulatory T cells, which determines the immune response. BRD4 participates in the differentiation of naïve CD4 T lymphocytes into Th17 cells (Cheung et al., 2017). Different BET inhibitors, such as MS417, JQ1, and I-BET762 can inhibit T-helper-cell differentiation by blocking the specific transcriptional activation of the specific factors. These iBETs blocked the transcription factor RORγT and inhibited IL-17 gene expression in Th17 cells, and inhibited TBX21 and INFγ gene in Th1 cells, but also had small effects on GATA3 and IL4 gene expression in Th2 cells (Cheung et al., 2017).

The role of Th17 cells and its main effector cytokine IL-17A in immune-mediated and chronic inflammatory renal diseases has increasing importance. Many experimental studies targeting Th17 immune response by different approaches, including neutralizing antibodies against IL17A or its soluble receptor, pharmacological inhibitors of RORऔT, as well as studies using genetically modified mice, have underscored the importance of IL17A in the pathogenesis of chronic inflammatory diseases, including immune and nonimmune renal damage (Bäckdahl et al., 2009; Edgerton et al., 2009; Pindjakova et al., 2012; Mele et al., 2013; Rodrigues-Díez et al., 2013; Mease et al., 2014; Park et al., 2014; Isailovic et al., 2015) Recently, we described that in an experimental model of diabetic nephropathy in BTBR ob/ob (leptin deficiency mutation) mice administration of an IL-17A neutralizing antibody, when renal dysfunction and structural alterations had already started, caused a beneficial effect, restoring renal damage parameters, mainly by inhibition of NF-κB/inflammation in the diabetic kidney (Lavoz et al., 2019). Moreover, treatments blocking IL-17A have shown beneficial effects in human clinical trials for the treatment of ankylosing spondylitis, chronic plaque psoriasis, and psoriatic arthritis and are currently tested in other inflammatory diseases (Leonardi et al., 2012; Baeten et al., 2013; Baeten et al., 2015; Mease et al., 2015). In two models of experimental renal damage, unilateral ureteral obstruction and immune mediated glomerulonephritis by nephrotoxic serum, we described that BET inhibition by JQ1 treatment markedly diminished the presence of IL17A expressing cells in the injured kidneys, associated with downregulation of renal levels of IL17A and other Th17-related cytokines, such as CCL-20 and CSF-1 (Suarez-Alvarez et al., 2017). These data suggest that BET inhibition could exert beneficial effects on renal damage by modulating the Th17 immune response (**Figure 3**). There are studies about the effect of BET pharmacological inhibition on different pathologies associated with Th17 response, results that support these data, as described in colitis (Cheung et al., 2017), experimental autoimmune encephalomyelitis (Jahagirdar et al., 2017a), retinal inflammatory disease (Eskandarpour et al., 2017), and asthma (Nadeem et al., 2018).

Besides regulating Th17 differentiation, BET proteins can also modulate Th17 response by a direct effect on the transcription of the IL17A gene. BRD4 and BRD2 bind to the regulatory region of CNS2 that controls IL-17A transcription (Mele et al., 2013). The treatment with JQ1 in a model of renal damage, downregulated IL17A gene, showing that this mechanism is also operating in injured kidneys (Suarez-Alvarez et al., 2017). Moreover, in Th17 cells a transcriptional coactivator such as p300 was able to bind to the IL-17A gene promoter supplying easier access to chromatin (Wang et al., 2012). All data suggest that BET inhibition could be an additional therapeutic option to block the inflammatory effects mediated by IL-17A in the kidney.

### BET Proteins in Renal Fibrosis

Several preclinical studies s suggest that iBETs can also present antifibrotic properties, as described in pulmonary (Tang et al., 2013), liver (Ding et al., 2015), cardiac (Spiltoir et al., 2013), and renal (Xiong et al., 2016; Zhou et al., 2017; Wang et al., 2018) fibrosis. In a mouse model of bleomycin-induced lung fibrosis, mouse treated with JQ1 had less collagen I deposition compared to control values (Tang et al., 2013). In this study the evaluation of lung fibroblasts isolated from patients with idiopathic pulmonary fibrosis (IPF) or healthy donors demonstrated that JQ1 ameliorated the phenotypic changes induced by the disease, such as increased cellular migration and proliferation. They found that JQ1 inhibited the secretion of IL-6, and they described an association between Ac-H4K5 and Brd4 involved in IL-6 gene regulation, therefore suggesting that iBET-mediated phenotypic changes could be mediated by IL-6 regulation (Tang et al., 2013). In a carbon tetrachloride (CCl4) mouse model of liver injury, the animals injected with JQ1 presented lower fibrosis and diminished expression of markers of hepatic stellate cell activation. Moreover, the *in vitro* evaluation of three different BET inhibitors (JQ1, I-BET-151, and PFI-1) showed a decrease of gene expression of fibrotic and hepatic stellate cell activation markers, such as type 1 collagen, α-smooth muscle actin (α-SMA), and desmin. In addition, JQ1 pretreatment abolished TGF-beta-induced fibroblast-like morphology changes in cultured hepatic stellate cell (Ding et al., 2015). (Spiltoir et al., 2013) showed that cardiac hypertrophy was significantly reduced by JQ1 in primary neonatal rat ventricular myocytes treated with phenylephrine or phorbol-12-myristate-13-acetate, determined by changes in atrial natriuretic factor levels. The *in vivo* effect of JQ1 on cardiac remodeling was demonstrated in a transverse aortic constriction model. JQ1 treatment reduced left ventricular systolic dysfunction, atrial natriuretic factor expression levels and interstitial fibrosis (Spiltoir et al., 2013). Other studies also confirm the beneficial effect of JQ1 in fibrosis and inflammation consequent to the radiation used in radiotherapy in thoracic cancer, by suppressing BRD4, c-MYC, p65 activation collagen I, TGF-β, and phosphorylated-SMAD2/3 after irradiation (Jung et al., 2015). Other BET inhibitor, RVX-297, specific for BD2, also diminished fibrosis in experimental models of arthritis (Jahagirdar et al., 2017b).

In the experimental model of UUO-induced renal damage several studies have observed that treatment with different iBETs, I-BET151, and JQ1, diminished renal fibrosis (Xiong et al., 2016; Zhou et al., 2017). However, as commented before, JQ1 also exert anti-inflammatory effects in UUO model, as renal inflammation occurs earlier than ECM accumulation, the amelioration of renal fibrosis described in this model could be subsequent to the inhibition of renal inflammation. Hypertensive nephropathy is one of the most common secondary nephropathies that trigger fibrosis in the kidney. A study performed in human kidney biopsies from hypertensive nephropathy patients and in an experimental of hypertension induced by systemic administration of AngII, described that BRD4 protein levels were upregulated in human and mouse kidney samples. Mice treatment of JQ1 diminished AngII-induced kidney fibrosis (Wang et al., 2019). However, as commented in the UUO model, previous studies have found that JQ1 treatment also diminished renal inflammation after 3 days of AngII administration (Suarez-Alvarez et al., 2017). Future studies should be done to study the direct effect of iBETs in experimental renal fibrosis, to clearly elucidate whether these compounds could block or even reverse the progression of renal fibrosis, independently of their anti-inflammatory effects. In fact, in all the experimental studies iBET treatment was started before renal damage occurs, and there is a lack of therapeutic interventions.

Several *in vitro* data have demonstrated direct antifibrotic effects of iBETs in renal cells. Studies using the cell line NRK-49F of rat renal fibroblasts showed that I-BET151 inhibited cell activation and proliferation, measured by the expression levels of α-SMA, and diminished ECM proteins overexpression, including collagen I and fibronectin (Xiong et al., 2016). Accordingly, BRD4 gene silencing inhibited TGF-β1 induced renal fibroblasts activation (Zhou et al., 2017). Moreover, *in vitro* studies in tubular epithelial cells stimulated with TGF-β1, showed that BRD4 inhibition, by gene silencing or treatment with JQ1, downregulated the expression of fibrotic-related genes, such as α-SMA and fibronectin, collagen IV, and fibronectin (Zhou et al., 2017). In pathological conditions, tubular epithelial cells can be damaged, causing death, by apoptosis/necrosis, or if there is on sublethal damage leads to a phenotype conversion, named epithelial-to-mesenchymal transition (EMT). In the context of renal damage, partial EMT is characterized by phenotypic changes leading to the loss of epithelial cell properties, the acquisition of mesenchymal markers (such as α-SMA), and induction of an aberrant senescence–secretome characterized by the production of proinflammatory and profibrotic proteins, that can contribute to renal fibrosis (Lovisa et al., 2016). Therefore, pharmacological interventions targeting partial EMT could be used as antifibrotic treatments for renal diseases. *In vitro* studies have found that JQ1 restored EMT-related changes induced by TGF-β1 in tubular epithelial cells (Wang et al., 2018). Moreover, in activated tubular epithelial cells, JQ1 inhibited a large array of mediators, some of them belonging to senescence–secretome, as IL-6 (Suarez-Alvarez et al., 2017). In experimental fibrosis induced by AngII-induced renal damage JQ1 restored changes in EMT-related proteins (suppressed the expression of vimentin, but increased the expression of epithelial markers such as E-cadherin and Zo-1) (Wang et al., 2019). In other pathologies, BRD4 inhibition suppresses EMT, as described in breast cancer cells (Shi and Vakoc, 2014). NF-κB regulates genes involved in EMT, including snail family transcriptional repressor (Tian et al., 2018), showing another target of iBETs in the regulation of EMT. Cardiac fibrosis contributes to the pathogenesis of cardiovascular diseases through endothelial–mesenchymal transition (EndMT). In a model of transverse aortic constriction, BRD4 expression was upregulated in endothelial cells. In cultured endothelial cells the pharmacological or genic blockade of BRD4 by JQ1 or shRNAs, respectively, attenuated TGF-β-induced EndMT (Song et al., 2019a). These data show an important role of BRD4 in the modulation of phenotype conversion, and suggest that iBETs could act as anti-fibrotic drugs inhibiting partial EMT in renal injury.

**Figure 4:**
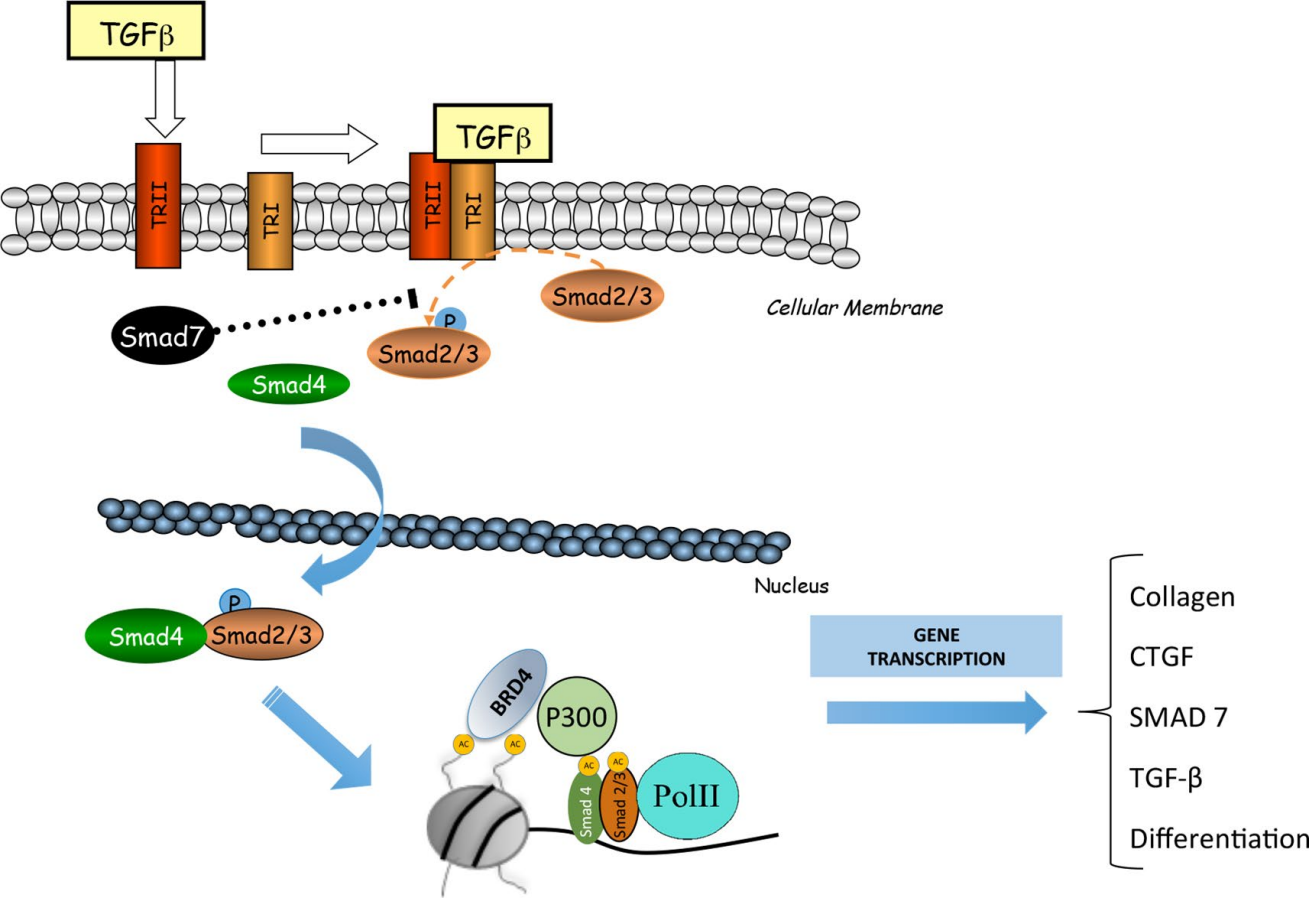
BRD4 is involved in the transforming growth factor β (TGF-ß)/SMAD signaling pathway. BRD4 interacts with the components of this signaling pathway to regulate the expression of genes related to the synthesis of extracellular matrix proteins (MEC) and fibrosis.

The Smad signaling pathway is the main mechanism involved in the regulation of fibrosis (Meng et al., 2016; Hu et al., 2018). Some authors linked the epigenetic molecule BRD4 with TGF-β/Smad signaling pathway (**Figure 4**). In a model of cardiac hypertrophy induced by aquatic physical training, transcriptomic analysis of JQ1 regulated showed downregulation of many genes under TGF-β control (Duan et al., 2017). In UUO model, mice treated with I-BET151 presented a downregulation in gene expression of α-SMA, a marker of activated miofibroblasts, and decreased fibronectin/collagen deposition in obstructed kidneys, associated to a diminution of phosphorylated-Smad3 expression whereas Smad7 expression levels were unchanged (Xiong et al., 2016)Therefore, TGF-β/Smad3 signaling deactivation might explain the antifibrotic effects of JQ1, *via* gene expression downregulating of several profibrotic factors. However, more detailed mechanistic experiments should be done to demonstrate the specific effect of iBET on the regulation of Smad pathway activation in renal diseases.

### iBETs as Potential Therapeutic Targets for Renal Diseases

The most important feature of chronic renal diseases is the functional deterioration of renal structures, and finally, the loss of renal function. Some experimental studies have investigated the effect of iBETs in renal function and structure. In a immune-model of progressive renal damage induced by glomerular antibasement membrane nephrotoxic serum, JQ1 treatment improved serum creatinine levels and urinary albumin to creatinine ratio, both biochemical parameters to evaluate renal function, and delayed the appearance of glomerular structural damage (extracapilary proliferation and fibrinoid necrosis) (Suarez-Alvarez et al., 2017). In another study in a model of acute kidney injury induced by the nephrotoxic cisplatin, JQ1 restored changes in renal function (as serum creatinine and BUN levels) and ameliorated renal lesions (hyaline cast formation and renal tubule lysis) (Sun et al., 2018b). In a model of autosomal dominant polycystic kidney disease in mouse strains with Pkd1 (pyruvate dehydrogenase kinase isozyme 1) mutations, JQ1 treatment strikingly delayed cyst growth and kidney enlargement, and preserved renal function through c-Myc–p21 signaling pathway modulation (Zhou et al., 2015). These studies demonstrated the beneficial effect of iBETs in the preservation of renal function in different experimental models of renal diseases.

### BET Proteins and Endothelial Dysfunction Associated to CVD–CKD

Endothelial dysfunction is one of the first events observed in CKD and cardiovascular disease (CVD) (Martens and Edwards, 2011). The loss of an adequate function of the endothelium, which regulates the interaction of cells and circulating proteins with the resident cells of the vascular wall, affects vascular homeostasis, thus promoting the beginning of the vascular damage (McCarron et al., 2019). As described above, iBETs regulate the inflammatory response. A study conducted in primary human pulmonary microvascular endothelial cells (HPMECs) from healthy donors showed that JQ1 decreased mRNA and protein levels of IL6 and IL8 and diminished the recruitment of p65 NF-κB to IL6 and IL8 promoters. The same study reported that the treatment of the cells with JQ1 inhibited the proliferation and migration of HPMECs and prevented its cell cycle progression (Mumby et al., 2017). In a model of acute lung inflammation and in HUVECs stimulated with TNF-α or LPS, JQ1 reduced the expression of inflammatory factors (IL-6 and IL-8) and adhesion molecules (ICAM-1, VCAM-1 and E-selectin) (Huang et al., 2017). However, there are no studies evaluating whether BET inhibition could directly, or not, modulate on vascular reactivity and functionality, including key mechanism involved in this process, such as the redox signaling or kinases activation.

### BET Proteins in Cardiovascular Pathologies as Subjacent Pathologies to CKD

Cardiovascular events are some of the main causes of death in CKD patients. There are several studies about the contradictory role of BET inhibitors in the improvement/impairment of the pathological features of heart failure (HF). Several data report that BRD4 is an important activator of gene transactivation during pathologies that lead to cardiac hypertrophy (Meng et al., 2014; Park-Min et al., 2014). A study performed in neonatal rat ventricular cardiomyocytes showed that several iBET I-BET-151, PFI-1 (a selective inhibidor against BRD4), and apabetalone reduced cardiomyocyte hypertrophy (Anand et al., 2013). In a model of transverse aortic constriction in mice, JQ1 attenuated multiple main features of heart failure by diminution of associated genes (Myh7, CTGF, Nppa, Nppb, and Rcan1) (Duan et al., 2017). In a model of ligation of the proximal left anterior descending coronary artery, BET inhibition ameliorated cardiomyocytes hypertrophy and fibrosis (Duan et al., 2017). However, there is one study showing the deleterious effect of BET inhibition, specifically IBET-151. In this study IBET-151 induced impairment of heart function determined by a reduced right and left ventricular fractional shortening and a decrease in the velocity-time-integral in the aorta and pulmonary artery, associated with induced structural and functional alterations of the heart mitochondria analyzed by transmission electron microscopy (Piquereau et al., 2019). These results suggest that more deeply studies will be necessary in the future in this field.

### BET Proteins in Vascular Calcification as a Secondary Effect of CKD

In CKD patients vascular calcification in a common complication characterized by important calcium phosphate deposits in the blood vessels walls. During this process, the vascular smooth muscle cells differentiated into osteoblasts, creating calcium deposits that compromise the elasticity of the vascular wall (Moe and Chen, 2004; Alves et al., 2014). There are several treatments for vascular calcification, such as inhibitors of phosphate binders or calcimimetics (Iyemere et al., 2006), but recently iBETs has been tested as a novel approach to this problem. Apabetalone has been shown to reduce serum alkaline phosphatase (ALP) in CKD patients with a history of cardiovascular events (Kulikowski et al., 2018). There are previous references about the role of ALP with adverse cardiovascular outcomes in CKD patients that normally developed vascular calcification (Haarhaus et al., 2017) (Taliercio et al., 2013). However, the role of ALP is complex and will require future investigations. A recent study has shown that apabetalone prevented matrix mineralization of cultured primary human coronary artery VSMCs by targeting ALP gene expression (Gilham et al., 2019). In this *in vitro* study, apabetalone through an epigenetic mechanism diminished several procalcific genes which drives vascular smooth muscle cell transdifferentiation (ALP and RUNX2). These authors demonstrate that BRD4 is redistributed on chromatin during transdifferentiation to alter gene expression and generate unique BRD4-rich enhancers associated with calcification (Gilham et al., 2019). However, the investigation of the involvement of iBETs in additional mechanism activated in vascular calcification, such as downregulation of the expression of genes involved in inflammation, or noncanonical WNT signaling, will require future investigations. There are some ongoing clinical trials that will give us information about the effects of iBETs on vascular calcification and other cardiovascular complications in CKD patients.

### BET Proteins and miRNAs Profile Associated

Functional roles for miRNAs have been described in numerous biological processes that include differentiation, development, cell proliferation, and apoptosis. (Erson & Petty, 2008; Stefani and Slack, 2008; Garzon et al., 2009). It is evident that miRNAs play a critical role in the maintenance of normal tissue homeostasis, so changes in their expression are of high relevance in pathological processes. Recent evidence indicates that miRNAs play a fundamental role in the regulation of cellular and molecular processes involved in renal and vascular diseases (Gebeshuber et al., 2013; Laffont & Rayner, 2017). An RNA sequencing study performed in endothelial cells showed miRNAs transcriptional profile changes induced by TNF-α that could be regulated by iBETs. TNF-α induced the upregulation of 44 miRNAs, such as mir 146a, mir-155, mir-455, mir-887, and mir-377. Interestingly, pretreatment of the cells with JQ1 downregulated TNF-α-mediated overexpression of mir-146a and mir-155. This study also demonstrated that BRD4 was recruited to the upstream gene loci of miR-146a and miR-155 and BRD4 gene deletion modulated pri-miR-146a and miR-155HG expression. In addition, using an inflammatory model induced by LPS, they found that mice treated with agomirs of miR-146a and miR-155 showed downregulation of inflammatory genes, such as VCAM-1, Selectin, and TRAF6, and decreased the number of circulating leukocytes (Duan et al., 2016). This study points that miR-146a and miR-155 play a key role in renal inflammation, and suggest that can be target of iBETs. A study in oligodendrocyte-precursor cells (OPCs) injured by regulating oxygen/glucose deprivation (OGD) showed that miR-146b-5p expression was reduced. The overexpression of miR-146b-5p induced cell growth and viability, and reduced the apoptosis and oxidative stress in OPCs submitted to OGD. In these cells BRD4 expression was diminished by miR-146b-5p. Moreover, the BRD4 silencing with a siRNA showed a protective effect in OGD-injured OPCs and modulated the Keap1/Nrf2/ARE signaling pathway (Li et al., 2019).

MiRNA-29 is a key regulator of pulmonary (Tang et al., 2019), liver (Huang et al., 2019), and renal fibrosis (Huang et al., 2019). The analysis of the miRNA signature linked to BET proteins in chronic obstructive pulmonary disease demonstrated that these patients presented a reduced miR-29b plasma levels associated with an increase of BRD4 levels. miR-29b levels in these patients were correlated with pulmonary function and inflammation and IL-8 expression (Tang et al., 2019). A study in streptozotocin-induced diabetic mice demonstrated that miR-29a overexpression by lentiviral infection acts as a positive regulator of Wnt/β-catenin signaling in mesangial cells and protects cell from apoptosis and fibrosis induced by diabetes (Hsu et al., 2016); but currently, there is no renal studies that link this specific miRNA with the modulation of BET proteins. Another study of cholestatic liver fibrosis induced by bile duct-ligation showed that mice overexpressed miR-29a decreased fibrosis (determined by α-SMA expression) and reduced the expression of hepatic BRD4 and SNAI1 in cholestatic livers. In hepatic stellated cells, miR-29a overexpression diminished the protein levels and gene expression of EZH2, MeCP2, and SNAI1 and increased PPAR-γ. Similarly, JQ1 reduced C-MYC, EZH2, and SNAI1 expression. Both, mimic miR-29a and JQ1 treatments, inhibited hepatic stellated cells migration and proliferation (Huang et al., 2019). There are several studies about the miRNA profile that regulate BET proteins in several oncological pathologies such as gastric cancer (Song et al., 2019b), cutaneous T-cell lymphomas (Kohnken et al., 2019), hepatocellular carcinoma (He et al., 2018), or prostate cancer (Guan et al., 2019). In a study performed in diffuse large B-cell lymphoma, cells treated with the iBET OTX015 revealed modifications in the expression levels of miR-92a-1-5p, miR-21-3p, miR-155-5p, and miR-96-5p. These miRNAs are involved in the modulation of different signaling pathways such as p53, apoptosis, MYC-targets, cell cycle regulation, B-cell receptor signaling, IL-6 signaling, the STAT3, PI3K, and NF-κB pathways (Mensah et al., 2018). Some of these miRNAs have a relevant role in renal pathology. Administration of an anti-miR-92a after crescentic rapidly progressive glomerulonephritis prevented albuminuria and kidney failure, by the modulation cyclin-dependent kinase inhibitor p57Kip2, indicating miR-92a inhibition as a potential therapeutic strategy for this renal disease (Henique et al., 2017). There are also some studies about the deleterious role of miR-92a related with vascular damage in CKD associated to endothelial dysfunction (Shang et al., 2017) or with the renal damage progression associated to atherosclerosis (Wiese et al., 2019). miR-155-5p is described as a key target of tubular renal cell injury in response to high glucose exposition *in vitro* modulating p53, sirt1 signaling pathway, and autophagy process (Wang et al., 2018). In relation with miR-21, there are several studies in experimental models of renal damage, diabetic nephropathy, or hypertension, that identified this miRNA as a key modulator of renal damage and fibrosis (Tang et al., 2019) (Song et al., 2018); (Wang et al., 2014); (Kölling et al., 2017); (Chen et al., 2017); (Hu et al., 2014) but there is no studies about its relationship with BET proteins in this field of study. Instead, a study in SW480 colon cancer mouse xenografts showed that treatment with JQ1 significantly downregulated miR-21 and reduced tumor growth and naked cuticle homolog 2 (Nkd2) expression and increased the apoptosis and β-catenin levels (Zhang et al., 2018). Another study in gastric cancer cells demonstrated that JQ1 reduced the proliferation and invasiveness of cancer cells through the induction of cellular senescence (increased cellular SA-β-Gal activity and elevated p21 levels). The authors also showed that p21 levels were modulated by miR-106b-5p. The overexpression of miR-106b-5p diminished JQ1-induced p21 expression and cellular senescence (Dong et al., 2018). All these results reveal that there is an extensive miRNA signature associated with the modulation of BET proteins in several proliferative disorders. In despite, there are not many studies about these proteins in the renal disease field. The modulation of BET proteins through miRNAs could be a therapeutic option to reverse the key pathological processes in the progression of renal disease such as the inflammatory response (modulating proinflammatory factors and inflammatory cell infiltration in the kidney), the apoptosis, oxidative stress, and fibrosis.

### iBETs in Clinical Trials

There are now more than 23 ongoing clinical trials evaluating the safety, the pharmacokinetics, and the pharmacodynamic effects of different iBETs in several pathologies. Variants of MK-8628/OTX-015 (MK-8628-002, MK-8628-003, MK-8628-005, MK-8628-006), a potent BET inhibitor specific to BRD2/3/4 developed by Oncoethix GmbH Corp and Merck Sharp & Dohme Corp., are currently tested in oncological diseases, such as advanced solid tumors, glioblastoma multiforme, and hematological malignancies (https://ClinicalTrials.gov/NCT02259114,NCT02296476¸NCT02698189,NCT02698176). An open-label, multicenter, dose-escalation phase 1/1b study in patients with acute myelogenous leukemia or non-Hodgkin lymphoma is analyzing a novel BET inhibitor FT-1101 (specific against BRD2/3/4 and BRDT. Forma Therapeutics, Inc) (Home - ClinicalTrials.gov. (n.d.), 2019) (https://ClinicalTrials.gov/NCT02543879). Another multicentric study (phase Ib) has set out to evaluate RO6870810/TEN-010, given as mono- and combination therapy to patients with advanced multiple myeloma (https://ClinicalTrials.gov/NCT03068351.Hoffmann-LaRoche). Other trials in progressive lymphoma and multiple myeloma patients studied the sequential dose escalation of BET inhibitor CPI-0610 (specific inhibitor against BD-1 of BRD2-4/BRDT¸ Constellation Pharmaceuticals) (https://ClinicalTrials.gov/NCT02157636).

**Figure 5:**
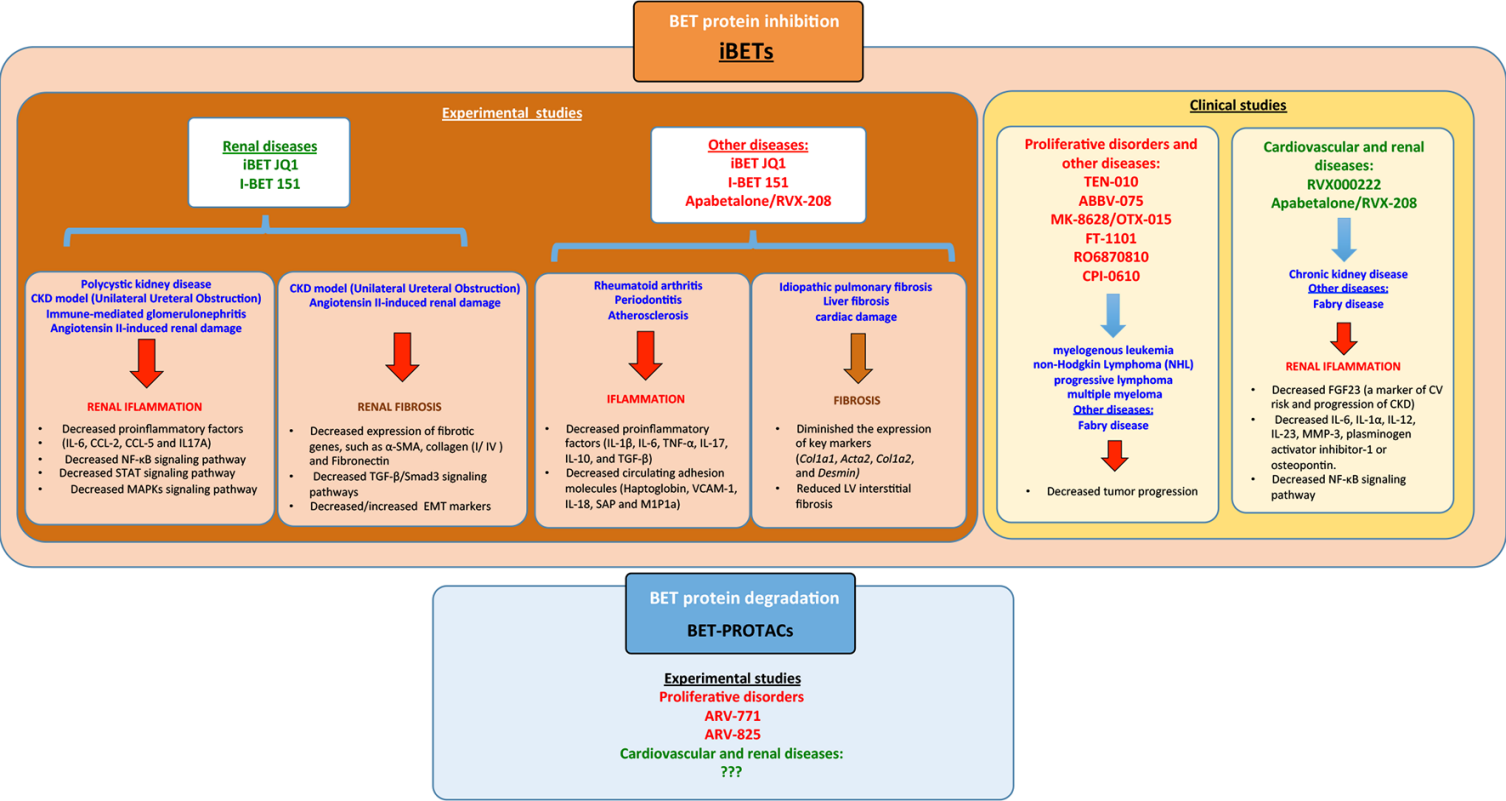
Summary of the different bromodomain and extraterminal (BET) modulators in experimental/clinical studies in the context of several diseases including renal disease and the signaling pathways associated. iBETs interact with several signaling pathways to regulate inflammation and fibrosis.

The BD2 selective inhibitor Apabetalone (RVX-208/RVX000222), developed by Resverlogix, has been evaluated in patients with diabetes mellitus type 2 and with cardiovascular diseases of high risk (https://ClinicalTrials.gov/NCT02586155). In a recent report, the bioinformatics analysis about the impact of apabetalone on the plasma proteome in patients with impaired kidney function, showed that this iBET could modulated key molecular pathways involved in immunity and inflammation, oxidative stress, endothelial dysfunction, vascular calcification, and coagulation (Wasiak et al., 2018). Fabry disease is a rare X-linked lysosomal storage disorder caused by mutations in the α-galactosidase A gene inducing severe complications, including cardiomyopathy and end-stage renal disease. There is an ongoing study by Resverlogix Corp. in patients with Fabry disease and CKD testing oral apabetalone administration on key biomarkers of vascular calcification, such as RANKL and osteoprotegerin, and key markers of inflammation. such as high-sensitivity C-reactive protein (https://ClinicalTrials.gov/NCT03228940). The results of these ongoing clinical trials will help us to understand if BET inhibitors can be used in patients with cardiovascular and renal diseases (**Figure 5**).

New IBETs, the BET-PROTACs, have been developed. These are proteolysis-targeting chimera proteins, that recruit and utilize an E3-ubiquitin ligase to effectively degrade BET proteins. The treatment of mantle cell lymphoma cells with two different BET-PROTACs (ARV-771 and ARV-825), targeting BRD4 or BRD2/3/4, respectively, showed more potent apoptotic effect compared to iBET OTX015 (MK-8628), an iBET that selectively blocks BRD2/3/4. ARV-771 treatment inhibited *in vivo* growth and induced greater survival improvement of immune-depleted mice engrafted with mantle cell lymphoma cells (Sun et al., 2018a). Other studies also described the role of BET-PROTACs in castration-resistant prostate cancer, using a cancer xenograft mouse model. In this model treatment with ARV-771, improved tumor progression and suppressed markers of poor prognosis, such as androgen receptor signaling (Raina et al., 2016).

The evaluation of preclinical studies and ongoing clinical trials revealed that, in some cases, treatment with BET-inhibitors presents adverse effects. An experimental study in mice developed by Bolden et al. showed that BRD4 blockade by reversible transgenic interference RNA (RNAi) has deleterious effects in several tissues, such as epidermal hyperplasia, alopecia, and decreased cellular diversity and stem cell depletion in the small intestine (Bolden et al., 2014). In several clinical trials with diverse iBETs (NCT02391480; NCT02516553; NCT01949883; NCT02296476; NCT01713582; NCT02683395), diverse side effects have been described, including gastric or intestinal located-toxicities (diarrhea, nausea, vomiting), fatigue, hyperbilirubinemia, thrombocytopenia, anemia, and in some cases kidney injury (Amorim et al, 2016; Berthon et al., 2016; Hottinger et al., 2016; Aftimos et al., 2017; Blum et al., 2018; Patnaik et al., 2018).

New IBETs, the BET-PROTACs, have been developed. These are proteolysis-targeting chimera proteins that recruit and utilize an E3-ubiquitin ligase to effectively degrade BET proteins. The new BET-PROTACs have modifications in the molecular structure of the linker region of the PROTAC to optimize the blockade of bromodomains, improved cellular permeability, tissue distribution, and metabolism (Wang et al., 2019). The treatment of mantle cell lymphoma cells with two different BET-PROTACs (ARV-771 and ARV-825), targeting BRD4 or BRD2/3/4, respectively, showed more potent apoptotic effect compared to iBET OTX015 (MK-8628), an iBET that selectively blocks BRD2/3/4. ARV-771 treatment inhibited *in vivo* growth and induced greater survival improvement of immune-depleted mice engrafted with mantle cell lymphoma cells (Sun et al., 2018a). Other studies also described the role of BET-PROTACs in castration-resistant prostate cancer, using a cancer xenograft mouse model. In this model treatment with ARV-771, result in improves tumor progression and a suppression of androgen receptor signaling which increased levels that are associated with a poor prognosis of the disease (Raina et al., 2016) (**Figure 5**).

Some clinical trials have shown a limited antitumor activity and secondary effects associated with cytotoxicity. These limitations suggest that new therapeutic approaches are needed to target BET proteins. Experimental studies have shown that BET inhibition versus BET degradation (BET-PROTACs) elicited different cellular effects and biological outcomes in proliferative disorders (Yang et al., 2019). These modifications of BET-PROTACs expected to have more profound effect on BET-mediated transcriptional complexes and turn these inhibitors in the actual best option to improve the limited clinical efficacy of BET inhibitors and expand the field of use to other pathologies such as renal damage, in which epigenetic modifications have an important role in the pathology progress.

## Conclusion and Perspectives

Emerging evidence remark the importance environment modifications in the origin of pathological features in CKD and in the cardiovascular related complications. Some key processes implicated in the progression of renal disease such as inflammation and fibrosis have demonstrated that could induce epigenetic modifications associated to the deterioration of pathological process, highlighting the potential of epigenetic therapeutic strategies against BET proteins for CKD treatment. Preclinical studies have proven beneficial effects of iBETs in acute and chronic renal damage. The anti-inflammatory effects, by direct inhibition of gene expression and NF-KB activation, and the antifibrotic effects, by the inhibition of cell proliferation and phenotype changes, are, at least in part, responsible of these beneficial effects. New generation of BET inhibitors or degraders, more specific and less toxic, have been synthetized and now are submitted to preclinical and clinical studies. Their future findings will help us to elucidate all the beneficial or deleterious effect in several pathologies, being necessary to include studies in renal pathology.

## Author Contributions

All the authors have written, reviewed the manuscript and approved the final version. MR-O has also contributed to financial support.

## Funding

This work was supported by grants from the Instituto de Salud Carlos III (ISCIII) and Fondos FEDER European Union (PI17/00119; Red de Investigación Renal REDINREN: RD16/0009 and PI17/01244), Sociedad Española de Nefrologia and “NOVELREN-CM: Enfermedad renal crónica: nuevas Estrategias para la prevención, Diagnóstico y tratamiento”; B2017/BMD-3751, Comunidad de Madrid. The “Juan de la Cierva Formacion” training program of the Ministerio de Economia, Industria y Competitividad supported the salary of SR-M (FJCI-2016-29050).

## Conflict of Interest

The authors declare that the research was conducted in the absence of any commercial or financial relationships that could be construed as a potential conflict of interest.

## Abbreviations

ET, extraterminal domain; iBETs, BET proteins inhibitors; NF-κB, nuclear factor-κB; UUO, unilateral ureteral obstruction; aa, Aminoacids; AAAs, abdominal aortic aneurysms; Acta2, α-smooth muscle actin; ALP, alkaline phosphatase; AngII, angiotensin II; ARA, II angiotensin II receptors type I antagonists; BAF, barrier to autointegration factor; BCL2, B-cell lymphoma 2; BET, bromo extraterminal; BRD2, bromodomain-containing protein 2; BRD3, bromodomain-containing protein 3; BRD4, bromodomain-containing protein 4; BRDT, bromodomain testis-specific protein; BTBR, black and tan, brachyuric; CCL-20, chemokine (C–C motif) ligand 20; CCL2, chemokine (C–C motif) ligand 2; CCl4, carbon tetrachloride; RPGN, crescentic rapidly progressive glomerulonephritis; CCL5/RANTES, regulated on activation, normal T expressed and secreted; CIA, collagen-induced arthritis; CKD, chronic kidney disease; COPD, chronic obstructive pulmonary disease; CSF-1, colony stimulating factor 1; CTD, c-terminal domain; CVD, cardiovascular disease; CXCL10, chemokine (C-X-C motif) ligand 10; CXCL11, chemokine (C-X-C motif) ligand 11; DNA, deoxyribonucleic acid; EMT, epithelial–mesenchymal transition; ERK1, extracellular signal-regulated kinases 1; ERK2, extracellular signal-regulated kinases 2; ESRD, end stage renal damage; GATA3, trans-acting T-cell-specific transcription factor 3; HDL, high density lipoprotein; HF, heart failure; HPMECs, human pulmonary microvascular endothelial cells; HSC, hepatic stellate cell; HUVECs, human umbilical cordon endothelial cells; IFNγ, interferon gamma; IL-10, interleukin-10; IL-17, interleukin-17; IL-18, interleukin-18; IL-1b, interleukin-1b; IL4, interleukin-4; IL6, interleukin-6; iNOS, inducible nitric oxide synthase; IPF, idiopathic pulmonary fibrosis; LDL, low density lipoprotein; LFs, lung fibroblast; LIF, leukemia inhibitory factor; LPS, lipopolysaccharide; LV, left ventricular; M1P1a, macrophage inflammatory protein 1α; MAECs, murine aortic endothelial cells; MAPK, mitogen-activated protein kinase kinase; MCAP, mitotic protein associated to cromatin; miRNA, small non-coding RNA molecule; MMP-9, matrix metalloproteinase 9; NFATc1, nuclear factor of activated T cell C1; Nkd2, naked cuticle homolog 2; OGD, oxygen/glucose deprivation; OPCs, oligodendrocyte-precursor cells; P-TEFb, positive elongation factor b; PE, phenylephrine; PI3K, phosphatidylinositol-3-kinase; RANKL, receptor activator of nuclear factor kappa-B ligand; *RORC,* RAR related orphan receptor C; SAP, synapse-associated protein; STAT3, signal transducer and activator of transcription 3; TBX21, T-box transcription factor 21; TGF-β, transforming growth factor β; TNF-α, tumor necrosis factor-α; TRAP, tartrate-resistant acid phosphatase; VCAM1, vascular cell adhesion molecule 1; α-SMA, alpha-smooth muscle actin.
